# Supplementary material for: Synthesis of FePcS–PMA–LDH Cointercalation Composite with Enhanced Visible Light Photo-Fenton Catalytic Activity for BPA Degradation at Circumneutral pH
Source: Materials (Basel). 2020 Apr 21;13(8):1951. doi: 10.3390/ma13081951 (PMC7215779; doi:10.3390/ma13081951)
Supplement: Supplementary file 1 [file materials-13-01951-s001.pdf]

materials

MDPI

Supplementary Materials

Synthesis of FePcS–PMA–LDH Cointercalation Composite with Enhanced Visible Light Photo-Fenton Catalytic Activity for BPA Degradation at Circumneutral pH

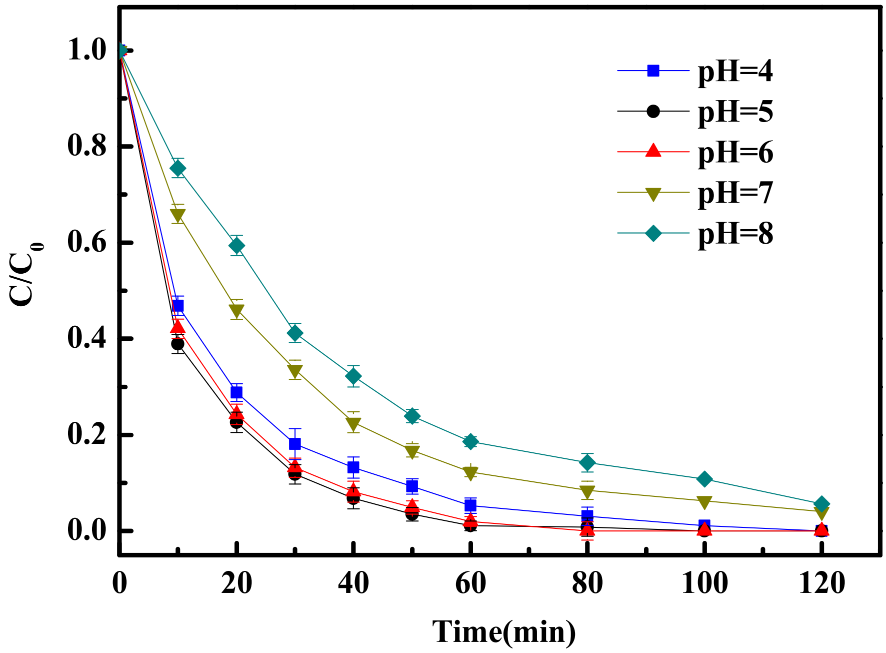

Figure S1. Effects of initial pH value on the degradation of BPA. ([BPA] = 10 mg/L; [H_2O_2] = 6 mM; catalyst dosage = 0.4 g/L; Light intensity = 500 W.).

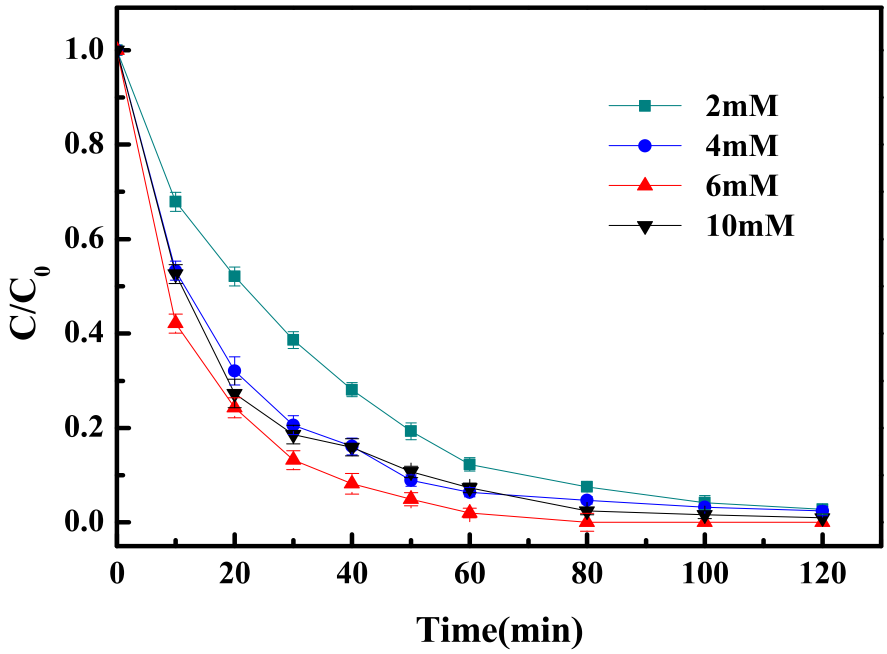

Figure S2. Effects of H_2O_2 dosages on the degradation of BPA. ([BPA] = 10 mg/L; pH = 6.0; catalyst dosage = 0.4 g/L; Light intensity = 500 W.).

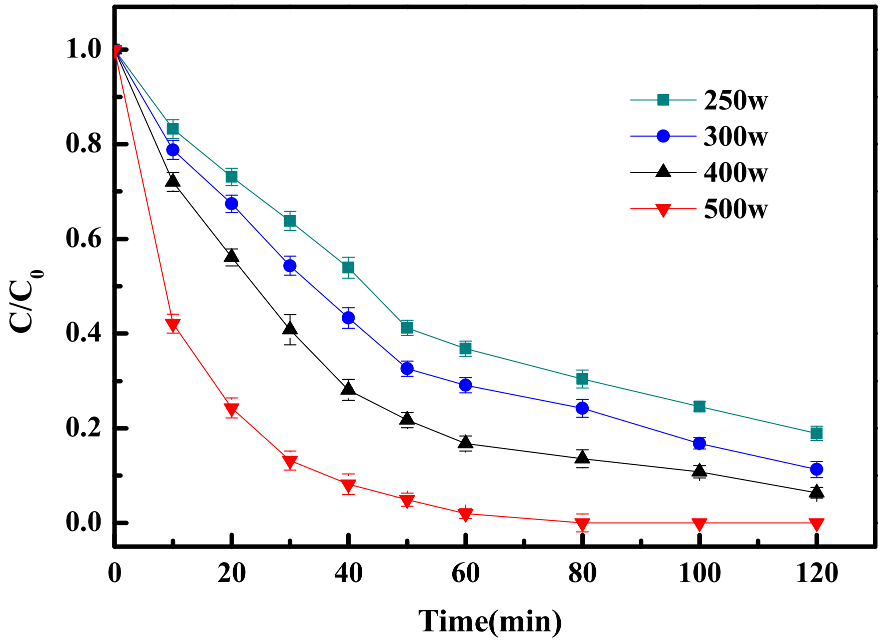

Figure S3. Effects of light intensity on the degradation of BPA. ([BPA] = 10 mg/L; [H_2O_2] = 6 mM; pH = 6.0; catalyst dosage = 0.4 g/L.).

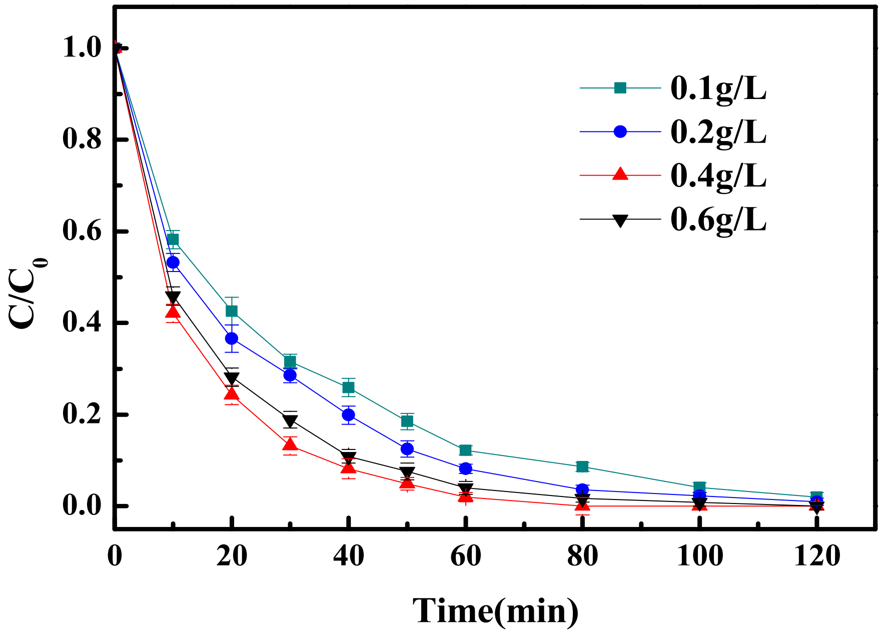

Figure S4. Effects of catalyst loading on the degradation of BPA. ([BPA] = 10 mg/L; [H_2O_2] = 6 mM; pH = 6.0; Light intensity = 500 W.).

1. Effects of pH

pH is one of the crucial factors in photo-Fenton process [1]. The effect of pH on BPA degradation in this system with FePcS-PMA-LDH was studied. As shown in Figure S1, the efficiency of BPA degradation increases when the pH decreases from 8 to 5 but then turns to decrease while further decreasing pH to 4. However, the BPA degradation efficiency in the system with pH 5 is close to that in pH = 6. Above results demonstrate that the catalyst FePcS-PMA-LDH can be applied in a relative

wide pH range. Considering the cost and practical application, pH 6 was considered as the optimum condition in this system.

2. Effects of H_2O_2 Dosage

It is known that the concentration of H_2O_2 is also an important parameter in photo-Fenton system. As illustrated in Figure S2, when the H_2O_2 dosage increases from 2 mM to 6 mM, the BPA degradation rate also increases. This result is because that higher H_2O_2 concentration can leads to more generation of •OH radicals. However, a further increase of the H_2O_2 concentration result in a lower BPA removal rate, which results from the scavenging effect of •OH radicals by excess H_2O_2 [2]. Hence, the optimal H_2O_2 concentration in this system is decided as 6mM**.**

3. Effects of irradiation intensity

Four irradiation intensities (250 W, 300 W, 400 W and 500 W) were set up to explore the influence of irradiation intensity on BPA degradation (as shown in Figure S3). The results reflect that the BPA degradation is increased while increasing the intensity of light illumination. This result can be explained by the fact that higher light intensity results in more photo energy for activation of catalyst and thus leads to more active species**.** Therefore, 500 W is considered as the optimum irradiation intensity in the system.

4. Effects of Catalyst Dosage

Figure S4 exhibits the effect of catalyst loading on BPA degradation. When increasing the catalyst loading from 0.1 g/L to 0.4 g/L, the BPA removal rate is increased as well. However, the removal BPA efficiency is slightly decreased when catalyst loading reaches to 0.6 g/L. This is owing to that the excess catalyst particles leads to the decreased visible light penetration [3]. Therefore, the optimum catalyst loading in this system is 0.4g/L.

Reference:

1. Li, R.; Gao,Y.; Jin, X.; Chen, Z.; Megharaj, M.; Naidu, R. Fenton-like oxidation of 2,4-DCP in aqueous solution using iron-based nanoparticles as the heterogeneous catalyst. *J. Colloid Interface Sci.* **2015**, *438*, 87–93.
2. Ahmed, B.; Limem, E.; Abdel-Wahab, A.; Nasr, B. Photo-Fenton treatment of actual agro-industrial wastewaters. *Ind. Eng. Chem. Res.* **2011**, *50*, 6673–6680.
3. Daneshvar, N.; Salari, D.; Khataee, A.R. Photocatalytic degradation of azo dye acid red 14 in water on ZnO as an alternative catalyst to TiO_2. *J. Photochem. Photobiol. A Chem.* **2004**, *162*, 317–323.

© 2020 by the authors. Submitted for possible open access publication under the terms and conditions of the Creative Commons Attribution (CC BY) license (http://creativecommons.org/licenses/by/4.0/).
